# Robot-assisted laparoscopic surgery versus conventional laparoscopic surgery in randomized controlled trials: A systematic review and meta-analysis

**DOI:** 10.1371/journal.pone.0191628

**Published:** 2018-01-23

**Authors:** Hyunsuk Frank Roh, Seung Hyuk Nam, Jung Mogg Kim

**Affiliations:** 1 Department of Biomedical Science, Hanyang University College of Medicine and Graduate School of Biomedical Science and Engineering, Seoul, Korea; 2 Department of Thoracic and Cardiovascular Surgery, Hanyang University Guri Hospital, Guri, Gyunggi, Korea; 3 Department of Microbiology and Biomedical Science, Hanyang University College of Medicine and Graduate School of Biomedical Science and Engineering, Seoul, Korea; National Academy of Medical Sciences, NEPAL

## Abstract

**Importance:**

This review provides a comprehensive comparison of treatment outcomes between robot-assisted laparoscopic surgery (RLS) and conventional laparoscopic surgery (CLS) based on randomly-controlled trials (RCTs).

**Objectives:**

We employed RCTs to provide a systematic review that will enable the relevant community to weigh the effectiveness and efficacy of surgical robotics in controversial fields on surgical procedures both overall and on each individual surgical procedure.

**Evidence review:**

A search was conducted for RCTs in PubMed, EMBASE, and Cochrane databases from 1981 to 2016. Among a total of 1,517 articles, 27 clinical reports with a mean sample size of 65 patients per report (32.7 patients who underwent RLS and 32.5 who underwent CLS), met the inclusion criteria.

**Findings:**

CLS shows significant advantages in total operative time, net operative time, total complication rate, and operative cost (*p* < 0.05 in all cases), whereas the estimated blood loss was less in RLS (*p* < 0.05). As subgroup analyses, conversion rate on colectomy and length of hospital stay on hysterectomy statistically favors RLS (*p* < 0.05).

**Conclusions:**

Despite higher operative cost, RLS does not result in statistically better treatment outcomes, with the exception of lower estimated blood loss. Operative time and total complication rate are significantly more favorable with CLS.

## Introduction

Although conventional laparoscopic surgery (CLS) allows more rapid postoperative recovery and has superior cosmetic outcomes compared with open laparotomy, CLS has several technical drawbacks [1] including a limited range of motion of instruments and related loss of dexterity, fixed instrument tips, and an inadequate visual field associated with an unstable camera view and assistant traction [2]. The introduction of robot-assisted laparoscopic surgery (RLS) was expected to open new avenues through its potential to overcome the drawbacks of CLS as a result of better ergonomics and enhanced dexterity with tremor filtration, numerous instrumental tips for the EndoWrist instrument, and 3D optical systems [3, 4]. Due to these advances, there is encouraging emerging evidence supporting RLS as an alternative technique to CLS.

In spite of the aforementioned advantages of RLS there are several controversial aspects of this approach, such as operative time, blood loss, conversion rate, complication rate, length of hospital stay, and operative cost. Numerous studies have reported clinical outcomes with regard to these aspects, and meta-analyses have assessed the pros and cons of robotics in various departments, including surgery, urology, and gynecology [5–14]. However, robotics is a relatively new surgical approach and RCT data have only recently become commonly available. Thus, there is a limited number of RCTs on each surgical procedure [3], and the lack of robust clinical evidence has frequently been emphasized [4, 15]. On incorporating all surgical procedures; previous statistically nonsignificant results due to limited RCT evidence for individual surgical procedure, may become statistically significant because of greater statistical power.

The scope of meta-analyses exclusively on RCTs [1, 16, 17] considers only a specific surgical procedure to convey more homogenous statistical results, compared to the that of a meta-analysis across surgical procedures [18]. However, the intrinsic properties of robotic instruments throughout overall surgical procedures could be mistakenly reported as the results of the unique properties of the particular surgical procedure. In contrast, the scope of meta-analysis [18] has been enlarged to combine RCTs across surgical procedures. Nonetheless, this regrettably omitted, among others, cholecystectomy and did not present forest plots with subgroup analyses. The forest plots with subgroup analysis of each surgical procedure intuitively would embrace the need for further floods of separate meta-analysis for each surgical procedure and encourage the combined discussion on the surgical robotics across surgical procedures. Our effort for integrating the previous individual studies and meta-analyses aims to provide the basis to statistically weigh the effectiveness and efficacy of surgical robotic instruments in controversial fields on surgical procedures both overall and on each individual surgical procedure.

## Materials and methods

### Identification of studies

A systematic review of the literature was conducted by electronic search to find RCT studies with date of publication from 1981 to 2016 inclusively on PubMed, EMBASE, and Cochrane databases with the following search strategy: mention at least one of robot, robotic, robotics, robotically, robot-assisted, robotic-assisted and, at the same time, at least one of laparoscopic, laparoscopy, laparoscope, since both RLS and CLS should be discussed; RCT evidence with a keyword of at least one of randomized, randomised, random, and RCT. It should be noted that Web of Science (Core) and SCOPUS were additionally used to find the full text for the articles that were identified by the Excerpta Medical Database of EMBASE. Two independent researchers (HFR and SHN) extracted the following data from selected studies: references, study type, population characteristics, and relevant clinical outcomes. Discrepancies between the two authors were reviewed and resolved by the senior investigator (JMK). Protocol.io: http://dx.doi.org/10.17504/protocols.io.k7ucznw

### Eligibility criteria

The criteria for eligibility of evidence were as follows: the original article should present a RCT comparison between RLS and CLS in human subjects with basic demographic information, including at least one of the following aspects: total operative time (Total-OT), net operative time (Net-OT), estimated blood loss (EBL), number of transfusions (Transf), conversion rate (Conv), total complication rate (Total-Cx), intra-operative complication rate (Intra-Cx), post-operative complication rate (Post-Cx), length of hospital stay (LOHS), and total operative costs (Cost). Additionally, this study excluded RCT studies performed for simulation and training purposes.

### Statistical methods & bias

Cochrane Review Manager (version 5.3) enabled most of the statistical analysis, including bias assessments of RCTs, whereas the supplemental R script (S2 File) conveyed all the information to reproduce the results of Begg’s test and Egger’s test. A p-value < 0.05 was considered statistically significant. PRISMA checklist was employed as a protocol of meta-analysis and its guideline was followed [19]. Fixed-effects model was employed if the Higgin's I^2^ statistics were < 50%; otherwise, a random-effects model was applied. In addition to bias assessments on individual RCT evidence, publication biases were visually assessed based on funnel plots. Then, publication bias was numerically evaluated by Begg’s test [20] and Egger’s test [21], respectively, based on rank correlation and weighted linear regression.

### Handling operative outcomes

When extracting the numerical information from original articles, some do not report, for example, the standard deviations. If the relevant numerical values are missing from the original articles but reported in the meta-analysis, we copied the values from the corresponding meta-analysis. Otherwise, a request was sent to the corresponding author for the missing data. If the requested information was not available or was not received within 30 days after the request date, we imputed the missing standard deviations by taking the median value of the included studies [22].

#### Total-OT and Net-OT

If an article did not explicitly describe the method used to measure operative time, we regarded it as Total-OT. However, if an article distinguishes Total-OT from pure procedural time by, for example, skin-to-skin operative time, we treated it as Net-OT in this study.

#### Intra-Cx, Post-Cx, and Total-Cx

If an original article explicitly described whether the reported number of complications referred to either intra-operative or post-operative complication rates, we specifically reflected this. However, if the author did not make this distinction, we entered the number into the total complication rate (Total-Cx). In addition, Total-Cx was assumed to be the sum of intra-operative complication rate (Intra-Cx) and post-operative complication rate (Post-Cx), unless the original article [23] reported values for Intra-Cx and Post-Cx that did not add up to the reported Total-Cx.

#### Cost

The operative costs were divided by 1,000, since the forest plot of Cochrane Review Manager software does not seem to visually handle values greater than 1,000. However, the Z-values before and after the division by 1,000 were found to be the same numerically. If the operative costs were reported in currency other than US dollars, we indicated their currency.

## Results

### Literature search

**Fig 1:**
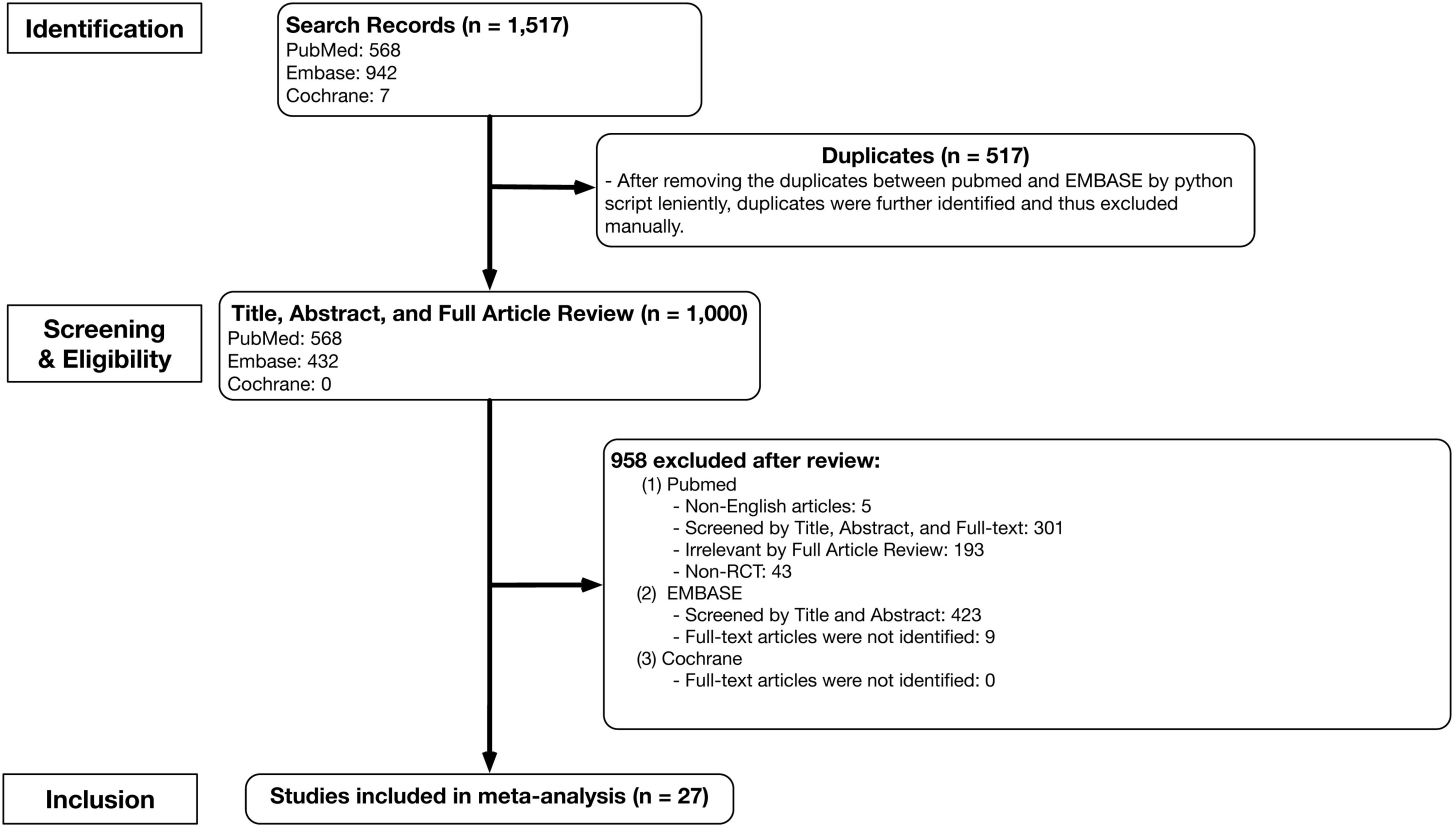
Schematic representation of the study selection process. This study attempted to screen leniently and perform a full-text search whenever possible in order to more confidently determine whether to include or exclude studies and extract information necessary for meta-analysis [50].

As illustrated in Fig 1, our search strategy identified 1,517 studies (568 from PubMed, 942 from EMBASE, and 7 from Cochrane) comparing treatment outcomes between RLS and CLS. After removing duplicate articles in the EMBASE database and Cochrane database that were also found in the PubMed database, a total of 1,000 articles (568 PubMed studies, 432 EMBASE studies, and 0 Cochrane studies) were examined. After reviewing the titles, abstracts, and full texts, 27 published studies [23–49] met all inclusion criteria and were included in the meta-analysis.

### Study characteristics

S1 Table lists the characteristics of all studies that compared the RCT treatment outcomes between RLS and CLS. Among the 27 RCT studies, with a mean sample size of 65 patients per study (32.7 patients for RLS and 32.5 patients for CLS), seven studies in gynecology, 15 in surgery, and five in urology were selected for inclusion in the analysis. With respect to gastrointestinal reconstruction studies, after gastrectomy, Sanchez *et al*. [48] restored continuation of the gastrointestinal tract via Roux-en-Y gastrojejunostomy. The type of robot employed in the included studies was the da Vinci system, with the exception of “MONA” by Cadiere *et al*. [29], “AESOP” by Aiono *et al*. [24] and Morino *et al*. [37], and “ZEUS-AESOP” by Nio *et al*. [41].

The previously described imputation methodology [22] was applied for the missing standard deviation of Total-OT [29, 34, 35, 37, 48], Net-OT [35], EBL [33–35, 38], LOHS [33–35, 37, 38, 41, 46, 48], and Cost [37, 38]. We had difficulty determining the number of patients allocated to RLS and CLS from one hysterectomy study [23], therefore the number of patients was taken from its meta-analysis [7]. Nakadi *et al*. reported two distinct Cost categories for robotic surgery as “Total” and “Total (with instrumentation costs in relation to the real OR occupation time: 4%)” [40]. For this study we chose to enter the latter as Cost, since the authors seemed to suggest that this was a more reasonable representation of the total robotic surgery cost. Three studies [38–40] reported Cost in Euros.

### Quality assessments

**Fig 2:**
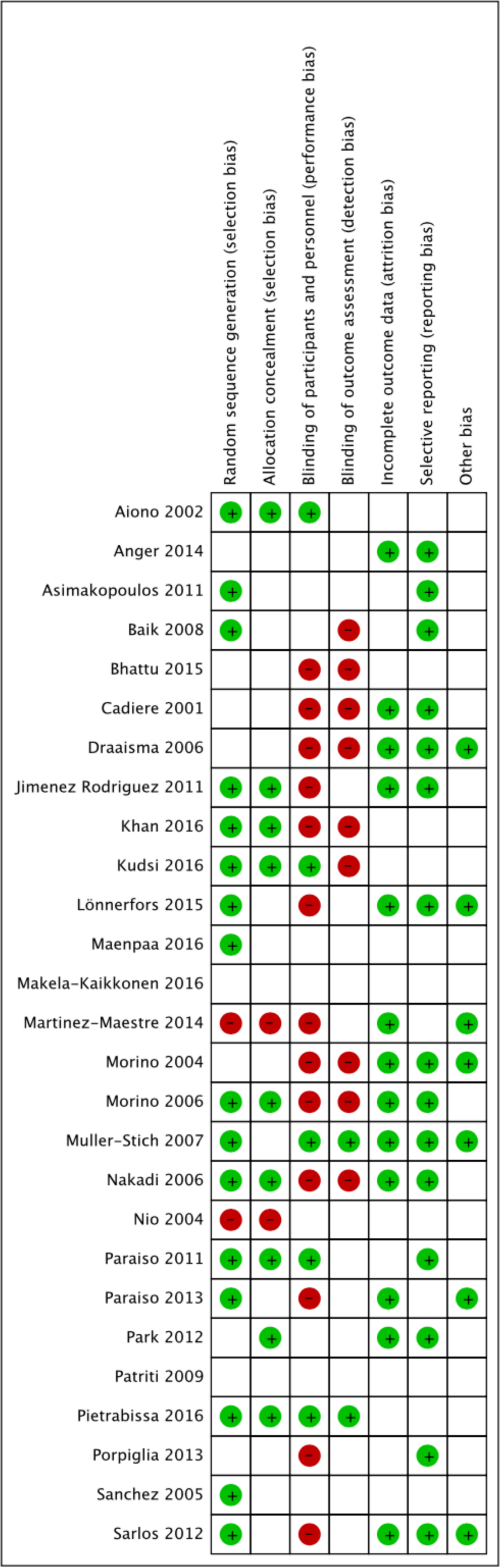
Risk of bias assessments. Bias assessments of RCTs were performed in terms of random sequence generation, allocation concealment, blinding of participants and personnel, blinding of outcome assessment, incomplete outcome data, selective reporting, and other biases. +, -, and a blank space denote lower risk, high risk, and unclear risk, respectively, for the risk judgement.

Bias assessments of RCTs in previous reviews [1, 16–18, 51–56] differed according to the judgements of the authors reviewing the same studies. Bias assessments of RCTs in previous reviews have been typically performed in terms of random sequence generation, allocation concealment, blinding of participants and personnel, blinding of outcome assessment, incomplete outcome data, selective reporting, and other biases. For example, the results of bias assessments of one colectomy study [27] for the aforementioned seven bias aspects were “+—?—? +?” in one meta-analysis [53] and “+ +—- + + -” in the other meta-analysis [1], where +, -, and? denote lower risk, high risk, and, unclear risk, respectively, for the risk judgement. This kind of discrepancy could be found frequently among meta-analyses. Thus, our quality assessments (Fig 2) were performed rather conservatively with a blank space for denoting unclear risk in consideration of previous assessments [1, 16–18, 51–56]. The risk of bias in the RCT was high and unclear overall.

### Treatment outcomes

**Fig 3:**
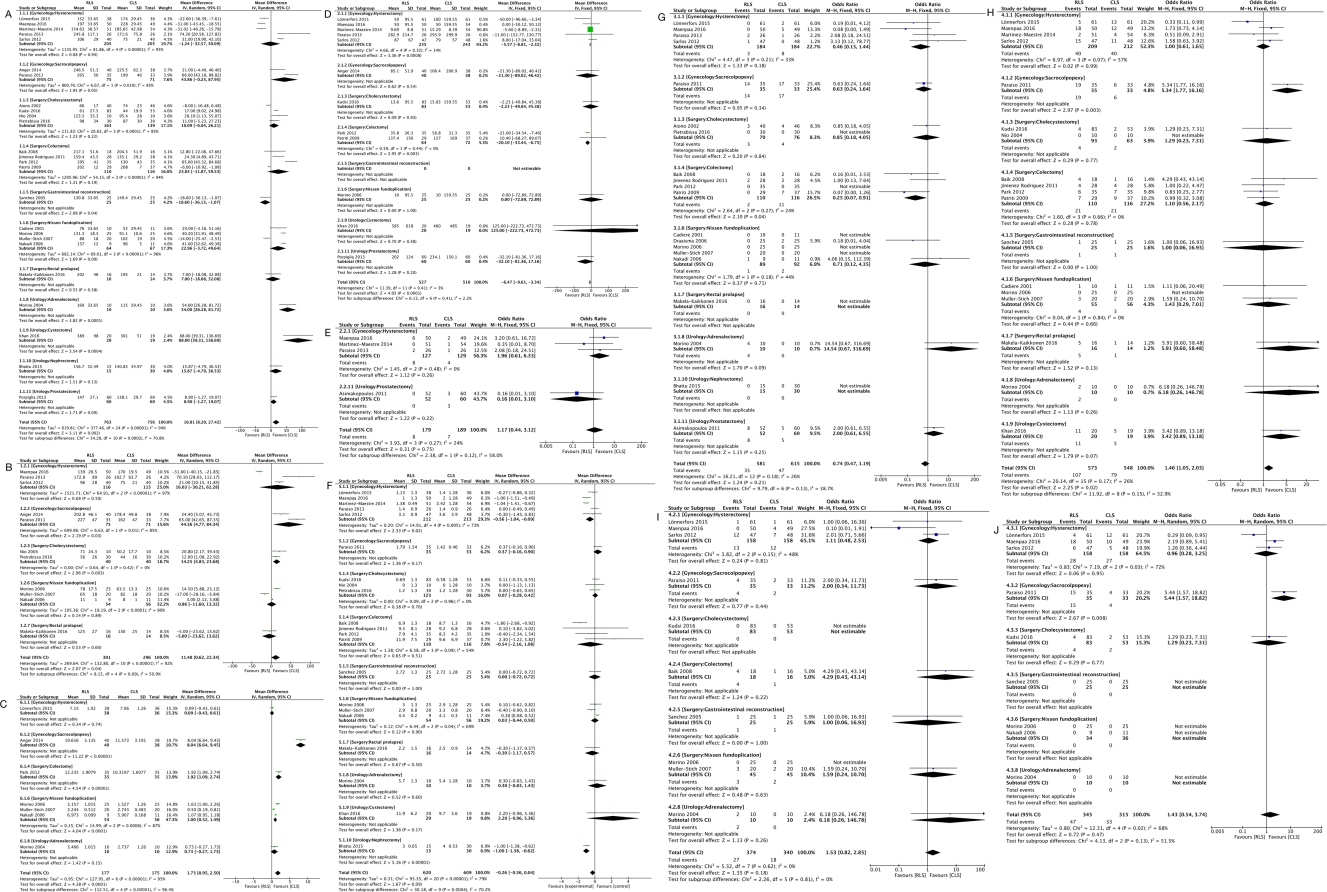
Forest plots with subgroups. Comparison of RLS and CLS with respect to (A) total operative time, (B) net operative time, (C) total operative cost, (D) estimated blood loss, (E) blood transfusion, (F) length of hospital stay, (G) conversion, (H) total complication, (I) intra-operative complication, and (J) post-operative complication. Note that, although Cochrane RevMan reports “Not estimable” for the study whose value include “0” value and does not take the study into account for the forest-plot analysis, the present study reports all available values from the studies for completeness. OR: odds ratio; WMD: weighted mean difference.

Fig 3 shows the results of comparison of the treatment outcomes between RLS and CLS. Since the Higgin's I^2^ was < 50%, the fixed-effect model was applied for EBL, Transf, Conv, Total-Cx, and Intra-Cx, whereas the random-effect model was employed for Total-OT, Net-OT, Post-Cx, LOHS, and Cost. Total-OT, Net-OT, Total-Cx, and Cost were statistically shown to favor CLS with pooled mean differences [95% confidence interval] of 16.81 [6.20, 27.42] with the high heterogeneity (P < 0.05, I^2^ = 94%) (Fig 3A), 11.48 [0.62, 22.34] with the high heterogeneity (P < 0.05, I^2^ = 92%) (Fig 3B), 1.46 [1.05, 2.03] with no evidence of observed heterogeneity (P = 0.17, I^2^ = 26%) (Fig 3H), and 1.73 [0.95, 2.50] with the high heterogeneity (P < 0.05, I^2^ = 95%) (Fig 3C), respectively, whereas EBL was shown to favor RLS with pooled mean difference of -6.47 and 95% CI of [-9.61, -3.34] with no evidence of observed heterogeneity (P = 0.41, I^2^ = 3%) (Fig 3D). No statistically significant difference between the groups was found for Transf, Intra-Cx, Post-Cx, or LOHS. With respect to forest plots on subgroups, Conv on colectomy and LOHS on hysterectomy statically favors RLS with pooled mean differences [95% confidence interval] of 0.25 [0.07, 0.91] with no evidence of observed heterogeneity (P = 0.27, I^2^ = 24%) (Fig 3G) and -0.56 [-1.04, -0.09] with the high heterogeneity (P < 0.05, I^2^ = 73%) (Fig 3F), respectively.

### Publication bias

Publication bias was assessed by funnel plots between the two groups. The funnel plots showed symmetric distributions indicating no evidence of publication bias among the studies (S1 Fig). In addition, with respect to Total-OT, there did not appear to be any strong evidence of publication bias from either Begg’s test (*p* = 0.2072) or Egger’s test (*p* = 0.2079) (S2 File).

## Discussion

This review provides a comprehensive comparison between RLS and CLS based on RCT studies in order to investigate the general trend of treatment outcomes for the controversial aspects of these approaches. Besides, the forest plots with subgroup analysis provide necessary information not only to investigate the comparison of treatment outcomes from the unique properties of the particular surgical procedure, but also to facilitate a more combined discussion from experts across various surgical procedures. From the statistical findings, it is concluded that despite its higher cost, RLS does not result in statistically improved treatment outcomes, with the exception of a lower EBL. Rather, Total-OT, Net-OT, and Total-Cx significantly favor CLS.

Because of the greater statistical power attained by integrating relevant RCT evidences, this study is able to report that, for example, Total-OT and Net-OT are significantly lower for CLS, although Liao *et al*. [10] and Albright *et al*. [16] reported that the operative time was insignificantly lower in CLS based on RCT evidence. Xiong *et al*. and Chuan *et al*. attributed the lower EBL of RLS to the use of robotic devices that provided better stability and avoided the damage caused by shaking hands in CLS and provided a greater field of view for detection of both large and small vessels [6, 57].

Clinical heterogeneity introduced by integrating various surgical procedures may contribute to the deviation from previous meta-analyses findings, presumably reflecting the intrinsic properties of each surgical procedure such as retroperitoneal involvement of prostatectomy [58]. The subgroup analyses favoring RLS on Conv on colectomy and LOHS on hysterectomy could be reasoned in association with properties of surgical robotics as follows: Lin *et al*. proposed that a lower Conv may be attributed to superior exposure and visualization of the operating field [3]. Ran *et al*. report that RLS was superior to CLS in terms of EBL and Conv, and proposed that the increased precision and dexterity of robotic platforms may contribute to these findings [7]. Chuan *et al*. reported that RLS was superior to CLS in terms of LOHS, and proposed that the more subtle operative technique and reduced level of invasiveness may be responsible for their findings [6]. Regarding Conv, surgeons might try a new surgical technology on a relatively less complicated surgery, which possibly distorts the outcome and results in inconsistent statistical significance. Thus, in consideration of the level of difficulties encountered in different surgeries, it may remain to be seen whether Conv would support RLS when more data are accumulated.

The present study stems from the motivation to contribute to the advancement in meta-analysis on surgical robotics by listing the limitations, followed by preliminary suggestions, if possible. Firstly, as pointed out by O’Neill *et al*. [4], some studies did not indicate explicitly whether the docking time for RLS was included in the operative time. The main cause of this confusion lies in the absence of standard ways of comparing operative time between the two groups. Some articles do not explicitly describe specifically how the RLS operative time—which can be broken into several pieces such as skin-to-skin, robot set-up time, etc.—was measured. Furthermore, although some original articles set strict rules for measuring operative time, unfortunately the terminology differs across studies, for example, "Operation time" [35], "Net operating time" [23], "Procedure" [25], “Dissection time” [41, 46], "skin-to-skin time" [38], "Effective operative time" [39], etc. These differences in terminology create additional difficulties in identifying the actual operative time. In this regard, for this study we chose to use “Net operative time,” which is intuitively different from “Total operative time”.

Secondly, the difference of indications across studies could have resulted in the inclusion of patients from each study variable. For example, in addition to the apparent discrepancy between the patient's conditions and clinical indications, not all the critical criteria of one study [58] could be possibly matched and compared against those of another study [47], due to insufficient information. In this regard, one study acknowledged that “[t]he different inclusion criteria and surgical techniques could potentially justify the differences between these results” [47]. Thus, please be aware of the limitation imposed by heterogeneities, which diminished the significance of the statistical results of weighing advantages and disadvantages.

Thirdly, none of the studies evaluated the degree of surgeon proficiency between RLS and CLS in the face of a new technology like robotics with an established technology like laparoscopy. According to Finkelstein *et al*., “to become proficient at [laparoscopic radical prostatectomy] or [robotic-assisted laparoscopic radical prostatectomy], […] a surgeon must perform anywhere from 8 to 12 cases to as many as 200 cases” [59]. In this regard, it may be worth taking into account surgeon experience, for example, number of surgeries, before comparing other aspects of a surgery.

Finally, whenever standard deviations were not available, imputations of the standard deviations were conducted. Although it is reported that “imputing missing standard deviations in meta-analyses can provide accurate results” [22], awareness of this limitation should be noted. It is proposed that future studies should be encouraged to report standard deviations whenever possible.

## Supporting information

S1 Fig Funnel plots. (A) total operative time, (B) net operative time, (C) total operative cost, (D) estimated blood loss, (E) blood transfusion, (F) length of hospital stay, (G) conversion, (H) total complication, (I) intra-operative complication, and (J) post-operative complication.
(ZIP)

S1 Table Patient demographic and clinical characteristics. Total-OT: total operative time, Net-OT: net operative time, Cost: total operative cost, EBL: estimated blood loss, Transf: number of transfusions, LOHS: length of hospital stay, Conv: conversion rate, Total-Cx: total complication rate, Intra-Cx: intra-operative complication rate, Post-Cx: post-operative complication rate.
(DOCX)

S1 File PRISMA checklist. (DOC)

S2 File R script for Begg’s test and Egger’s test. Tests were performed for total operative time.
(R) Click here for additional data file.
