# Supplementary material for: Robot-assisted laparoscopic surgery versus conventional laparoscopic surgery in randomized controlled trials: A systematic review and meta-analysis
Source: PLoS One. 2018 Jan 23;13(1):e0191628. doi: 10.1371/journal.pone.0191628 (PMC5779699; doi:10.1371/journal.pone.0191628)
Supplement: S1 Table — Total-OT: total operative time, Net-OT: net operative time, Cost: total operative cost, EBL: estimated blood loss, Transf: number of transfusions, LOHS: length of hospital stay, Conv: conversion rate, Total-Cx: total complication rate, Intra-Cx: intra-operative complication rate, Post-Cx: post-operative complication rate. (DOCX) [file pone.0191628.s002.docx]

**S1 Table. Patient demographic and clinical characteristics.**

| **Category** | | **First Author, Year** | **Country** | **Number of patients** | | **Study design** | **Treatment Outcomes** | | | | | | | | | |
| --- | --- | --- | --- | --- | --- | --- | --- | --- | --- | --- | --- | --- | --- | --- | --- | --- |
| **Domain** | **Name of Operation** |  |  | **RLS** | **CLS** |  | **Total-OT** | **Net-OT** | **EBL** | **Transf** | **Conv** | **Total-Cx** | **Intra-Cx** | **Post-Cx** | **LOHS** | **Cost** |
| **Gynecology** | **Hysterectomy** | Lönnerfors, 2015 [34] | Sweden | 61 | 61 | RCT | O |  | O |  | O | O | O | O | O | O |
|  |  | Maenpaa, 2016 [35] | Finland | 50 | 49 | RCT | O | O | O | O | O | O | O | O | O |  |
|  |  | Martinez-Maestre, 2014 [49] | Spain | 51 | 54 | RCT | O |  | O | O |  | O |  |  | O |  |
|  |  | Paraiso, 2013 [43] | USA | 26 | 26 | RCT | O | O | O | O | O |  |  |  | O |  |
|  |  | Sarlos, 2012 [23] | Switzerland | 47 | 48 | RCT | O | O | O |  | O | O | O | O | O |  |
|  | **Sacrocolpopexy** | Anger, 2014 [25] | USA | 40 | 38 | RCT | O | O | O |  |  |  |  |  |  | O |
|  |  | Paraiso, 2011 [42] | USA | 35 | 33 | RCT | O | O |  |  | O | O | O | O | O |  |
| **Surgery** | **Cholecystectomy** | Aiono, 2002[24] | UK | 40 | 46 | RCT | O |  |  |  | O |  |  |  |  |  |
|  |  | Kudsi, 2016[33] | USA | 83 | 53 | RCT | O |  | O |  |  | O | O | O | O |  |
|  |  | Nio, 2004 [41] | Netherland | 10 | 10 | RCT | O | O |  |  |  | O |  |  | O |  |
|  |  | Pietrabissa, 2016 [46] | Italy | 30 | 30 | RCT | O | O |  |  | O |  |  |  | O |  |
|  | **Colectomy** | Baik, 2008 [27] | Korea | 18 | 16 | RCT | O |  |  |  | O | O | O |  | O |  |
|  |  | Jimenez Rodriguez, 2011 [31] | Spain | 28 | 28 | RCT | O |  |  |  | O | O |  |  | O |  |
|  |  | Park, 2012 [44] | Korea | 35 | 35 | RCT | O |  | O |  | O | O |  |  | O | O |
|  |  | Patriti, 2009 [45] | Italy | 29 | 37 | RCT | O |  | O |  | O | O |  |  | O |  |
|  | **Gastrointestinal reconstruction** | Sanchez, 2005 [48] | USA | 25 | 25 | RCT | O |  |  |  |  | O | O | O | O |  |
|  | **Nissen fundoplication** | Cadiere, 2001 [29] | Belgium | 10 | 11 | RCT | O |  |  |  | O | O |  |  |  |  |
|  |  | Draaisma, 2006 [30] | Netherland | 25 | 25 | RCT |  |  |  |  | O |  |  |  |  |  |
|  |  | Morino, 2006 [38] | Italy | 25 | 25 | RCT | O | O | O |  | O | O | O | O | O | O |
|  |  | Muller-Stich, 2007 [39] | Germany | 20 | 20 | RCT | O | O |  |  | O | O | O |  | O | O |
|  |  | Nakadi, 2006 [40] | Belgium | 9 | 11 | RCT | O | O |  |  | O |  | O | O | O | O |
|  | **Rectal Prolapse** | Makela-Kaikkonen, 2016 [36] | Filand | 16 | 14 | RCT | O | O |  |  | O | O |  |  | O |  |
| **Urology** | **Adrenalectomy** | Morino, 2004 [37] | Italy | 10 | 10 | RCT | O |  |  |  | O | O | O | O | O | O |
|  | **Cystectomy** | Khan, 2016 [32] | UK | 20 | 19 | RCT | O |  | O |  |  | O |  |  | O |  |
|  | **Nephrectomy** | Bhattu, 2015 [28] | India | 15 | 30 | RCT | O |  |  |  | O |  |  |  | O |  |
|  | **Prostatectomy** | Asimakopoulos, 2011 [58] | Italy | 64 | 64 | RCT |  |  |  | O | O |  |  |  |  |  |
|  |  | Porpiglia, 2013 [47] | Italy | 60 | 60 | RCT | O |  | O |  |  |  |  |  |  |  |

Total-OT: total operative time, Net-OT: net operative time, EBL: estimated blood loss, Transf: number of transfusions, Conv: conversion rate, Total-Cx: total complication rate, Intra-Cx: intra-operative complication rate, Post-Cx: post-operative complication rate, LOHS: length of hospital stay, Cost: total operative cost.
